# Approximation formulas related to Somos’ quadratic recurrence constant

**DOI:** 10.1186/s13660-018-1859-8

**Published:** 2018-09-27

**Authors:** Bo Zhang, Chao-Ping Chen

**Affiliations:** School of Mathematics and Informatics, Henan Polytechnic University, Jiaozuo, China

**Keywords:** 40A05, 41A60, Somos’ quadratic recurrence constant, Asymptotic formula, Bell polynomials, Ordered Bell numbers

## Abstract

We present two classes of asymptotic expansions related to Somos’ quadratic recurrence constant and provide the recursive relations for determining the coefficients of each class of the asymptotic expansions by using Bell polynomials and other techniques. We also present continued fraction approximations related to Somos’ quadratic recurrence constant.

## Introduction

Somos [1] (see [2, p. 446] and [3]) defined the sequence

$$ g_{0}=1,\qquad g_{n}=ng_{n-1}^{2}, \quad n \in\mathbb{N}:=\{1,2,3,\ldots \}. $$

The first few terms are

$$ g_{0}=1,\qquad g_{1}=1, \qquad g_{2}=2, \qquad g_{3}=12, \qquad g_{4}=576, \qquad g_{5}=1\text{,}658\text{,}880, \qquad \ldots. $$

The following asymptotic expansion is known in the literature:

1.1 $$\begin{aligned} g_{n}&\sim \frac{\sigma^{2^{n}}}{n} \biggl(1+ \frac{2}{n}-\frac{1}{n^{2}}+\frac {4}{n^{3}}-\frac{21}{n^{4}}+ \frac{138}{n^{5}}-\frac{1091}{n^{6}}+\cdots \biggr)^{-1}, \end{aligned}$$

where

1.2 $$ \sigma=\sqrt{1\sqrt{2\sqrt{3\cdots}}}=\prod _{n=1}^{\infty }n^{1/2^{n}}=1.66168794 \ldots $$

is known as Somos’ quadratic recurrence constant. Formula (1.1) was proved by Somos, and it is cited in Finch’s book [2, p. 446] as Somos’ result. Note that the coefficient of $1/n^{5}$ in Finch’s book is 137, but actually it is incorrect and its correct value is 138 (see Weisstein, Eric W. “Somos’s Quadratic Recurrence Constant.” From MathWorld–A Wolfram Web Resource [3]). The constant *σ* appears in important problems from pure and applied analysis, it has motivated a large number of research papers (see, for example, [4–17]).

Nemes [15] studied the coefficients in the asymptotic expansion (1.1) and developed recurrence relations. More precisely, Nemes [15, Theorem 1] proved that

1.3 $$ g_{n}\sim \frac{\sigma^{2^{n}}}{n} \biggl(a_{0}+ \frac{a_{1}}{n}+\frac {a_{2}}{n^{2}}+\frac{a_{3}}{n^{3}}+\cdots \biggr)^{-1}, $$

where the coefficients $a_{k}$ (for $k\in\mathbb{N}_{0}:=\mathbb{N}\cup\{0\}$) are given by the recurrence relation

$$ a_{0}=1, \qquad a_{1}=2, \qquad a_{2}=-1, \qquad a_{k}=\sum_{j=1}^{k-1} \biggl((-1)^{k-j}\binom {k-3}{k-j}a_{j}-a_{k-j}a_{j} \biggr)\quad\text{for } k\geq3. $$

The coefficients $a_{k}$ also satisfy the following recurrence relation [15, Theorem 3]:

1.4 $$ a_{0}=1,\qquad a_{k}=\frac{1}{k}\sum _{j=1}^{k}(-1)^{j-1}2b_{j}a_{k-j} \quad\text{for } k\in\mathbb{N}, $$

where $b_{k}$ are the ordered Bell numbers defined by the exponential generating function [18, p. 189]

1.5 $$ \frac{1}{2-e^{x}}=\sum_{k=0}^{\infty} \frac{b_{k}}{k!}x^{k}. $$

The ordered Bell numbers $b_{k}$ are given explicitly by the formula

$$ b_{k}=\sum_{j=0}^{\infty} \frac{j^{k}}{2^{j+1}}. $$

The first few ordered Bell numbers are

$$ \begin{gathered} b_{0}=1,\qquad b_{1}=1, \qquad b_{2}=3,\qquad b_{3}=13,\qquad b_{4}=75,\\ b_{5}=541, \qquad b_{6}=4683,\qquad \ldots. \end{gathered} $$

Nemes [15, Theorem 2] proved that the generating function $A(x)=\sum_{k=0}^{\infty}a_{k}x^{k}$ of the coefficients $a_{k}$ has the following representation:

1.6 $$ A(x)=\exp \Biggl(\sum_{k=1}^{\infty} \frac {(-1)^{k-1}2b_{k}}{k}x^{k} \Biggr). $$

Chen [5, Theorem 2.1] presented a class of asymptotic expansions related to Somos’ quadratic recurrence constant, which includes formula (1.1) as its special case. Let $r\neq0$ be a given real number. The sequence $g_{n}$ has the following asymptotic formula:

1.7 $$\begin{aligned} g_{n}= \frac{\sigma^{2^{n}}}{n} \biggl(1+\frac{c_{1}}{n}+ \cdots+\frac {c_{j}}{n^{j}}+\cdots \biggr)^{-1/r}\quad\text{as } n\to\infty \end{aligned}$$

with the coefficients $c_{j}\equiv c_{j}(r)$ ($j = 1, 2, \ldots, m$) given by

1.8 $$\begin{aligned} c_{j}=(-1)^{j}\sum _{k_{1}+2k_{2}+\cdots+jk_{j}=j}\frac{(-2r)^{k_{1}+k_{2}+\cdots +k_{j}}}{k_{1}!k_{2}!\cdots k_{j}!} \biggl(\frac{b_{1}}{1} \biggr)^{k_{1}} \biggl(\frac{b_{2}}{2} \biggr)^{k_{2}}\cdots \biggl(\frac{b_{j}}{j} \biggr)^{k_{j}}, \end{aligned}$$

where $b_{k}$ ($k \in\mathbb{N}$) denotes the ordered Bell numbers and the summation in (1.8) is taken over all nonnegative integers $k_{1}, k_{2}, \ldots, k_{j}$ satisfying the equation $k_{1}+2k_{2}+\cdots+jk_{j}=j$.

The first aim of the present paper is to give recursive relations for determining the coefficients $c_{j}$ in (1.7) (Theorem 2.1). The second aim of the present paper is to establish a more general result, which includes expansion (1.7) as its special case (Theorem 2.2). Our last aim in this paper is to present continued fraction approximations related to Somos’ quadratic recurrence constant (Theorems 3.1 and 3.2).

## Asymptotic expansions

Theorem 2.1 below gives recursive relations for determining the coefficients $c_{j}$ in (1.7) by using the Bell polynomials.

The Bell polynomials, named in honor of Eric Temple Bell, are a triangular array of polynomials given by (see [19, pp. 133–134] and [20, 26])

2.1 $$ \begin{aligned}[b] & B_{n, k}(x_{1}, x_{2}, \ldots, x_{n-k+1}) \\ &\quad =\sum\frac{n!}{j_{1}!j_{2}!\cdots j_{n-k+1}!} \biggl(\frac{x_{1}}{1!} \biggr)^{j_{1}} \biggl(\frac {x_{2}}{2!} \biggr)^{j_{2}}\cdots \biggl(\frac{x_{n-k+1}}{(n-k+1)!} \biggr)^{j_{n-k+1}}, \end{aligned} $$

where the sum is taken over all sequences $j_{1}, j_{2}, j_{3}, \ldots, j_{n-k+1}$ of nonnegative integers such that

$$ \begin{aligned} & j_{1}+j_{2}+ \cdots+j_{n-k+1}=k\quad\text{and}\quad j_{1}+2j_{2}+ \cdots+(n-k+1)j_{n-k+1}=n. \end{aligned} $$

The sum

2.2 $$ B_{n}(x_{1}, x_{2}, \ldots, x_{n})=\sum_{k=1}^{n}B_{n, k}(x_{1}, x_{2}, \ldots, x_{n-k+1}) $$

is sometimes called the *n*th complete Bell polynomial. The complete Bell polynomials satisfy the following identity:

2.3 Bn(x1,x2,…,xn)=|x1(n−11)x2(n−12)x3(n−13)x4(n−14)x5⋯⋯xn−1x1(n−21)x2(n−22)x3(n−23)x4⋯⋯xn−10−1x1(n−31)x2(n−32)x3⋯⋯xn−200−1x1(n−41)x2⋯⋯xn−3000−1x1⋯⋯xn−40000−1⋯⋯xn−5⋮⋮⋮⋮⋮⋱⋱⋮00000⋯−1x1|.

In order to contrast them with complete Bell polynomials, the polynomials $B_{n, k}$ defined above are sometimes called partial Bell polynomials. The complete Bell polynomials appear in the exponential of a formal power series

2.4 $$ \exp \Biggl(\sum_{n=1}^{\infty} \frac{x_{n}}{n!}u^{n} \Biggr)=\sum_{n=0}^{\infty} \frac{B_{n}(x_{1}, \ldots, x_{n})}{n!}u^{n}. $$

The Bell polynomials are quite general polynomials and they have been found in many applications in combinatorics. Comtet [19] devoted much to a thorough presentation of the Bell polynomials in the chapter on identities and expansions. For more results, the reader is referred to [21, Chap. 11] and [22, Chap. 5].

### Theorem 2.1

*Let* *r* *be a given nonzero real number*. *Then the sequence* $g_{n}$ *has the following asymptotic expansion*:

2.5 $$\begin{aligned} g_{n}&\sim \frac{\sigma^{2^{n}}}{n} \Biggl(\sum _{k=0}^{\infty}\frac {c_{k}(r)}{n^{k}} \Biggr)^{-1/r} \quad\textit{as } n\to\infty, \end{aligned}$$

*with the coefficients* $c_{k}(r)$ (*for* $k \in\mathbb{N}_{0}$) *given by the recursive relation*

2.6 $$ c_{0}=1\quad\textit{and}\quad c_{k}(r)= \frac{2r}{k}\sum_{\ell=0}^{k-1}(-1)^{k-\ell-1}b_{k-\ell }c_{\ell}(r), $$

*where* $b_{k}$ (*for* $k \in\mathbb{N}_{0}$) *denotes the ordered Bell numbers defined by* (1.5).

### Proof

From (1.3), it follows that

2.7 $$\begin{aligned} \biggl(\frac{\sigma^{2^{n}}}{ng_{n}} \biggr)^{r}\sim A^{r}(1/n). \end{aligned}$$

On the other hand, from the definition of $A(x)$, it follows that

2.8 $$\begin{aligned} A^{r}(1/n)=\sum_{k=0}^{\infty} \frac{c_{k}(r)}{n^{k}}\quad\text{as } n\to\infty, \end{aligned}$$

where $c_{k}(r)$ (for $k \in\mathbb{N}_{0}$) are real numbers to be determined. By using (1.6) and (2.4), we have

$$ \begin{aligned} A^{r}(1/n)&=\exp \Biggl(\sum _{k=1}^{\infty}\frac{(-1)^{k-1}2rb_{k}}{k }\frac{1}{n^{k}} \Biggr) \\ &=\exp \Biggl(\sum_{k=1}^{\infty} \frac {(-1)^{k-1}(k-1)!2rb_{k}}{k!}\frac{1}{n^{k}} \Biggr) \\ &=\sum_{k=0}^{\infty}\frac{B_{k} (2rb_{1},-2rb_{2}, \ldots, (-1)^{k-1}(k-1)!2rb_{k} )}{k!} \frac{1}{n^{k}}. \end{aligned} $$

Therefore it is seen that the $c_{k}(r)$ in (2.8) can be expressed in terms of the Bell polynomials

2.9 $$ c_{k}(r)=\frac{B_{k} (2rb_{1},-2rb_{2},\ldots, (-1)^{k-1}(k-1)!2rb_{k} )}{k!}. $$

Bulò et al. [23, Theorem 1] proved that the complete Bell polynomials can be expressed using the following recursive formula:

$$ B_{k}(x_{1}, x_{2}, \ldots, x_{k})= \textstyle\begin{cases} \sum_{\ell=0}^{k-1}\binom{k-1}{\ell}x_{k-\ell}B_{\ell}(x_{1}, x_{2}, \ldots, x_{\ell}) & \text{if}\quad k \in\mathbb{N},\\ 1& \text{if} \quad k=0. \end{cases} $$

Thus, formula (2.9) can be rewritten as

$$\begin{aligned}& c_{0}=1\quad\text{and} \\& \begin{aligned} c_{k}(r)&=\frac{1}{k!}\sum _{\ell=0}^{k-1}\binom{k-1}{\ell }(-1)^{k-\ell-1}(k- \ell-1)!2rb_{k-\ell} \\ &\quad{}\times B_{\ell}\bigl(2rb_{1},-2rb_{2}, \ldots, (-1)^{\ell-1}(\ell-1)!2rb_{\ell}\bigr) \\ &=\frac{1}{k!}\sum_{\ell=0}^{k-1} \binom{k-1}{\ell}(-1)^{k-\ell -1}(k-\ell-1)!2rb_{k-\ell} \ell!c_{\ell}(r) \\ &=\sum_{\ell=0}^{k-1}\frac{(-1)^{k-\ell-1}2rb_{k-\ell}}{k }c_{\ell}(r) \quad\text{for } k\in\mathbb{N}. \end{aligned} \end{aligned}$$

The proof of Theorem 2.1 is complete. □

### Remark 2.1

The representation using a recursive algorithm for the coefficients $c_{j}$ in (1.7) is more practical for numerical evaluation than the expression in (1.8). We can directly calculate $c_{k}(r)$ in (2.9) by using identity (2.3).

### Remark 2.2

We find that a special case of (2.5) when $r=1$ yields immediately the asymptotic formula (1.1). Here, taking $r=-1$ and $-1/2$ in (2.5), respectively, we give two explicit expressions

2.10 $$\begin{aligned} g_{n}&\sim \frac{\sigma^{2^{n}}}{n} \biggl(1- \frac{2}{n}+\frac{5}{n^{2}}-\frac {16}{n^{3}}+\frac{66}{n^{4}}- \frac{348}{n^{5}}+\cdots \biggr)\quad\text{as } n\to\infty \end{aligned}$$

and

2.11 $$\begin{aligned} g_{n}&\sim \frac{\sigma^{2^{n}}}{n} \biggl(1- \frac{1}{n}+\frac{2}{n^{2}}-\frac {6}{n^{3}}+\frac{25}{n^{4}}+\cdots \biggr)^{2}\quad\text{as } n\to\infty. \end{aligned}$$

Theorem 2.2 establishes a more general result, which includes Theorem 2.1 as its special case.

### Theorem 2.2

*Let* *r* *be a given nonzero real number and* *m* *be a given nonnegative integer*. *Then the sequence* $g_{n}$ *has the following asymptotic expansion*:

2.12 $$\begin{aligned} g_{n}&\sim \frac{\sigma^{2^{n}}}{n} \Biggl(\sum _{k=0}^{\infty}\frac {d_{k}(r,m)}{n^{k}} \Biggr)^{-n^{m}/r} \quad\textit{as } n\to\infty, \end{aligned}$$

*with the coefficients* $d_{k}(r,m)$ (*for* $k \in\mathbb{N}_{0}$) *given by the recursive relation*

2.13 $$ d_{0}=1\quad\textit{and}\quad d_{k}(r,m)= \frac{r}{k}\sum_{j=1}^{k-m} \frac{(-1)^{j-1}2b_{j}(j+m)}{j}d_{k-m-j}(r, m), $$

*where* $b_{k}$ (*for* $k \in\mathbb{N}_{0}$) *denotes the ordered Bell numbers defined by* (1.5).

### Proof

From (2.5), it follows that

2.14 $$\begin{aligned} A^{r}(1/n)\sim \Biggl(\sum _{k=0}^{\infty}\frac{d_{k}(r, m)}{n^{k}} \Biggr)^{n^{m}} \quad\text{as } n\to\infty, \end{aligned}$$

where $d_{k}(r, m)$ (for $k \in\mathbb{N}_{0}$) are real numbers to be determined.

Taking the logarithm of (2.14) and applying (1.6) yields

$$\begin{aligned} r \Biggl(\sum_{k=1}^{\infty}\frac{(-1)^{k-1}2b_{k}}{k} \frac {1}{n^{k+m}} \Biggr)\sim\ln \Biggl(\sum_{k=0}^{\infty} \frac{d_{k}(r, m)}{n^{k}} \Biggr)\quad\text{as } n\to\infty. \end{aligned}$$

Replacing *n* by *x* gives

$$\begin{aligned} r \Biggl(\sum_{k=1}^{\infty}\frac{(-1)^{k-1}2b_{k}}{k} \frac {1}{x^{k+m}} \Biggr)\sim\ln \Biggl(\sum_{k=0}^{\infty} \frac{d_{k}(r, m)}{x^{k}} \Biggr). \end{aligned}$$

Differentiating each side with respect to *x* yields

$$\begin{aligned} r \Biggl(\sum_{k=0}^{\infty}\frac{d_{k}(r, m)}{x^{k}} \Biggr) \Biggl(\sum_{k=1}^{\infty} \frac {(-1)^{k-1}2b_{k}(k+m)}{k}\frac{1}{x^{k+m+1}} \Biggr)\sim\sum_{k=1}^{\infty} \frac{kd_{k}(r, m)}{x^{k+1}}. \end{aligned}$$

Hence,

$$ \begin{aligned} kd_{k}(r, m)=r\sum _{j=1}^{k-m}\frac{(-1)^{j-1}2b_{j}(j+m)}{j}d_{k-m-j}(r, m) \end{aligned} $$

and formula (2.13) follows. The proof of Theorem 2.2 is complete. □

### Remark 2.3

Setting $(r, m)=(-1, 1)$ and $(r, m)=(1, 1)$ in (2.12), respectively, we give two explicit expressions

2.15 $$\begin{aligned} g_{n}&\sim \frac{\sigma^{2^{n}}}{n} \biggl(1- \frac{2}{n^{2}}+\frac{3}{n^{3}}-\frac {20}{3n^{4}}+\cdots \biggr)^{n}\quad \text{as } n\to\infty \end{aligned}$$

and

2.16 $$\begin{aligned} g_{n}&\sim \frac{\sigma^{2^{n}}}{n} \biggl(1+ \frac{2}{n^{2}}-\frac{3}{n^{3}}+\frac {32}{3n^{4}}-\frac{87}{2n^{5}}+\cdots \biggr)^{-n}\quad \text{as } n\to\infty. \end{aligned}$$

### Remark 2.4

Setting $c_{k}(r):=d_{k}(r, 0)$, we obtain from (2.13) that

2.17 $$ c_{0}=1\quad\text{and}\quad c_{k}(r)= \frac{r}{k}\sum_{j=1}^{k}(-1)^{j-1}2b_{j}c_{k-j}(r). $$

It is easy to see that (2.6) is equivalent to (2.17). Setting $a_{k}:=c_{k}(1)$, (2.17) becomes (1.4).

## Continued fraction approximations

We define the sequence $(u_{n})_{n\in\mathbb{N}}$ by

3.1 $$ u_{n}=\frac{ng_{n}}{\sigma^{2^{n}}}- \biggl(1+\frac{a}{n+b+\frac {c}{n+d+\frac{p}{n+q}}} \biggr). $$

We are interested in finding fixed parameters *a*, *b*, *c*, *d*, *p*, and *q* such that $(u_{n})_{n\in\mathbb{N}}$ converges as fast as possible to zero. This provides the best approximations of the form

3.2 $$ g_{n}\approx\frac{\sigma^{2^{n}}}{n} \biggl(1+ \frac{a}{n+b+\frac {c}{n+d+\frac{p}{n+q}}} \biggr). $$

Our study is based on the following lemma, which is useful for accelerating some convergences, or in constructing some better asymptotic expansions.

### Lemma 3.1

([24, 25])

*If the sequence* $(\lambda_{n})_{n\in\mathbb{N}}$ *converges to zero and if the following limit*

$$ \lim_{n\to\infty}n^{k}(\lambda_{n}- \lambda_{n+1})=l\in\mathbb{R}, \quad k>1 $$

*exists*, *then*

$$ \lim_{n\to\infty}n^{k-1}\lambda_{n}= \frac{l}{k-1},\quad k>1, $$

*where* $\mathbb{R}$ *denotes the set of real numbers*.

### Theorem 3.1

*Let the sequence* $(u_{n})_{n\in\mathbb{N}}$ *be defined by* (3.1). *Then*, *for*

3.3 $$ a=-2,\qquad b=\frac{5}{2}, \qquad c=-\frac{7}{4}, \qquad d=\frac{69}{14},\qquad p=-\frac{376}{49},\qquad q= \frac{5171}{658}, $$

*we have*

3.4 $$ \lim_{n\to\infty}n^{8}(u_{n}-u_{n+1})=- \frac{158\text{,}319}{47}\quad \textit{and}\quad\lim_{n\to\infty}n^{7}u_{n}=- \frac{22\text{,}617}{47}. $$

*The speed of convergence of the sequence* $(u_{n})_{n\in\mathbb{N}}$ *is given by the order estimate* $O (n^{-7} )$ *as* $n\to\infty$.

### Proof

First of all, we write the difference $u_{n}-u_{n+1}$ as the following power series in $n^{-1}$:

3.5 $$\begin{aligned} u_{n}-u_{n+1}&=-\frac{a+2}{n^{2}}+\frac{12+a+2ab}{n^{3}}- \frac {65-3ac+a+3ab+3ab^{2}}{n^{4}} \\ &\quad{}+\frac{382+a-8abc-4acd+4ab-6ac+6ab^{2}+4ab^{3}}{n^{5}} \\ &\quad{}- \bigl(2587-5acd^{2}+a+5acp-15cab^{2}-20abc-10acd-10abcd \\ &\quad{}+5ab-10ac+10ab^{2}+10ab^{3}+5ac^{2}+5ab^{4} \bigr)\frac {1}{n^{6}} \\ &\quad{}+ \bigl(20\text{,}600-15acd^{2}+a+15acp-45cab^{2}-40abc-20acd \\ &\quad{}-30abcd-18cdab^{2}+12abcp-12abcd^{2}+6acpq+12acdp \\ &\quad{}+6ab-15ac+15ab^{2}+20ab^{3}+12ac^{2}d+18abc^{2}-24cab^{3} \\ &\quad{}-6acd^{3}+15ac^{2}+15ab^{4}+6ab^{5} \bigr)\frac{1}{n^{7}} \\ &\quad{}- \bigl(192\text{,}649-35acd^{2}+a+35acp-105cab^{2}-70abc-7ac^{3}+7acpq^{2} \\ &\quad{}+42c^{2}dab+21apcd^{2}-28cdab^{3}+21ab^{2}cp-21ab^{2}cd^{2}-14abcd^{3} \\ &\quad{}-14ac^{2}p+42c^{2}ab^{2}-35cab^{4}-35acd-70abcd-63cdab^{2} \\ &\quad{}+42abcp-42abcd^{2}+21acpq+42acdp+7ab-21ac+21ab^{2} \\ &\quad{}+35ab^{3}+42ac^{2}d+63abc^{2}-84cab^{3}-21acd^{3}+35ac^{2} \\ &\quad{}+35ab^{4}+21ab^{5}-7acp^{2}-7acd^{4}+21c^{2}d^{2}a+14abcpq \\ &\quad{}+14acdpq+7ab^{6}+28abcdp \bigr)\frac{1}{n^{8}} +O \biggl( \frac {1}{n^{9}} \biggr). \end{aligned}$$

The fastest sequence $(u_{n})_{n\in\mathbb{N}}$ is obtained when the first six coefficients of this power series vanish. In this case

$$ a=-2,\qquad b=\frac{5}{2}, \qquad c=-\frac{7}{4}, \qquad d=\frac{69}{14},\qquad p=-\frac{376}{49},\qquad q= \frac{5171}{658}, $$

we have

$$ u_{n}-u_{n+1}=-\frac{158\text{,}319}{47n^{8}}+O \biggl( \frac{1}{n^{9}} \biggr). $$

Finally, by using Lemma 3.1, we obtain assertion (3.4) of Theorem 3.1. □

Solution (3.3) provides the best approximation of type (3.2),

3.6 $$ g_{n}\approx\frac{\sigma^{2^{n}}}{n} \biggl( 1+ \frac{-2}{n+\frac{5}{2}+\frac{-\frac{7}{4}}{n+\frac {69}{14}+\frac{-\frac{376}{49}}{n+\frac{5171}{658}}}} \biggr). $$

Now we define the sequence $(v_{n})_{n\in\mathbb{N}}$ by

3.7 $$ v_{n}=\frac{ng_{n}}{\sigma^{2^{n}}}-\exp \biggl(\frac{a_{1}}{n+b_{1}+\frac {c_{1}}{n+d_{1}+\frac{p_{1}}{n+q_{1}+\frac{r_{1}}{n+s_{1}}}}} \biggr). $$

We are interested in finding fixed parameters $a_{1}$, $b_{1}$, $c_{1}$, $d_{1}$, $p_{1}$, $q_{1}$, $r_{1}$, and $s_{1}$ such that $(v_{n})_{n\in\mathbb{N}}$ converges as fast as possible to zero. This provides the best approximations of the form

3.8 $$ g_{n}\approx\frac{\sigma^{2^{n}}}{n}\exp \biggl( \frac{a_{1}}{n+b_{1}+\frac {c_{1}}{n+d_{1}+\frac{p_{1}}{n+q_{1}+\frac{r_{1}}{n+s_{1}}}}} \biggr). $$

Following the same method used in the proof of Theorem 3.1, we can prove Theorem 3.2, we omit it.

### Theorem 3.2

*Let the sequence* $(v_{n})_{n\in\mathbb{N}}$ *be defined by* (3.7). *Then*, *for*

3.9 $$ \begin{aligned} &a_{1}=-2,\qquad b_{1}=\frac{3}{2}, \qquad c_{1}=-\frac{25}{12}, \qquad d_{1}=\frac{219}{50}, \\ & p_{1}=- \frac{15\text{,}653}{1875},\qquad q_{1}=\frac {5\text{,}676\text{,}423}{782\text{,}650},\qquad r_{1}=-\frac{645\text{,}255\text{,}151\text{,}929}{34\text{,}302\text{,}297\text{,}260},\\ & s_{1}= \frac {113\text{,}583\text{,}705\text{,}304\text{,}934\text{,}619}{11\text{,}222\text{,}420\text{,}992\text{,}382\text{,}930}, \end{aligned} $$

*we have*

3.10 $$ \lim_{n\to\infty}n^{10}(v_{n}-v_{n+1})=- \frac {34\text{,}622\text{,}675\text{,}505\text{,}712\text{,}426\text{,}801}{175\text{,}652\text{,}791\text{,}358\text{,}450} $$

*and*

3.11 $$ \lim_{n\to\infty}n^{9}(v_{n}-v_{n+1})=- \frac {34\text{,}622\text{,}675\text{,}505\text{,}712\text{,}426\text{,}801}{1\text{,}580\text{,}875\text{,}122\text{,}226\text{,}050}. $$

*The speed of convergence of the sequence* $(v_{n})_{n\in\mathbb{N}}$ *is given by the order estimate* $O (n^{-9} )$ *as* $n\to\infty$.

Solution (3.9) provides the best approximation of type (3.8)

3.12 $$ g_{n}\approx\frac{\sigma^{2^{n}}}{n}\exp \biggl( \frac{-2}{n+\frac {3}{2}+\frac{-\frac{25}{12}}{n+\frac{219}{50}+\frac{-\frac {15\text{,}653}{1875}}{n+\frac{5\text{,}676\text{,}423}{782\text{,}650}+\frac{-\frac {645\text{,}255\text{,}151\text{,}929}{34\text{,}302\text{,}297\text{,}260}}{n+\frac {113\text{,}583\text{,}705\text{,}304\text{,}934\text{,}619}{11\text{,}222\text{,}420\text{,}992\text{,}382\text{,}930}}}}} \biggr). $$

## Conclusions

In this paper, we give asymptotic expansions related to the generalized Somos’ quadratic recurrence constant (Theorems 2.1 and 2.2). We present continued fraction approximations related to Somos’ quadratic recurrence constant (Theorems 3.1 and 3.2).
